# 

**Published:** 1851-09

**Authors:** 


### CONTENTS

### OF NO. III.

---

### ORIGINAL COMMUNICATIONS.

### ESSAYS AND CASES.

ART. I.—Clinical Observations in Private Practice. By E. B. HASKINS, M.D., of Clarksville, Tenn. - - 185

ART. II.—A Case of Strangulated Hernia, in which an operation was necessary. By W. J. OWEN, M.D., of Port Gibson, Miss. - - - - - 191

ART. III.—Pharmacy of the Greeks and Romans. Translated from the French of M. Cass: by WM. H. COBB, M.D., of Louisville, Ky. - - - - 192

ART. IV.—Remarkable migrations of a Pin and Needle through the Body of a Young Lady. By NAPOLEON B. ANDERSON, M.D , of Louisville, Ky. - - - - 200

ART. V.—Remarks on Dysentery. By WM. KENNEY, M.D., of Millersburgh, Ky. - - - - - 201

### REVIEWS.

ART. VI.—A Practical Treatise on the Diseases and Injuries of the Urinary Bladder, the Prostate Gland, and the Urethra. By S. D. GROSS M.D., Professor of Surgery in the University of Louisville, Member of the American Medical Association, author of Elements of Pathological Anatomy, etc. etc. - - - - 206

ART. VII.—Description of fifteen new Species of Crinoidea from the Sub-carboniferous Limestone of Iowa, collected during the United States Geological Survey of Iowa, Wisconsin, and Minnesota, in the years 1848–'9. By DAVID D. OWEN, M.D., and B. F. SHUMARD. M.D. 238

Art. VIII.—The Outlines of General Pathology. By M. L. Linton, M.D., Professor of the Theory and Practice of Medicine in the Medical Department of the St. Louis University. - - - - - 243

SELECTIONS FROM FOREIGN AND HOME JOURNALS.

Occlusion of the Vagina relieved by an operation. - 245
Hepatico-renal Circulation. - - - - - 247
Physiology of Women. - - - - 247
Chloroform in Gonorrhea. - - - - - 250
Cod-Liver Oil in Scrofulous Affections and in Consumption 250
Case of Gun-Shot Wound of the Spine. - - - - 255
Case of Popliteal Aneurism Cured by compression over the Tumor. - - - - - - 257
A case of Retrocession of the Cutaneous Efflorescence in Rubeola. 259
Remarks on the Cooking and Preserving of Meat. - 262
Trial for Alleged Rape, - - - - - 264

ORIGINAL INTELLIGENCE.

Cholera in Louisville. - - - - - - 269
Medical Convention. - - - - - - 273
Medical Society of Tennessee. - - - - - 275
University of Louisville. - - - - - 276
Circular American Medical Association. - - - 276

